# Osteosarcoma is characterised by reduced expression of markers of osteoclastogenesis and antigen presentation compared with normal bone

**DOI:** 10.1038/sj.bjc.6605723

**Published:** 2010-06-15

**Authors:** L Endo-Munoz, A Cumming, S Sommerville, I Dickinson, N A Saunders

**Affiliations:** 1 The University of Queensland, Diamantina Institute for Cancer, Immunology and Metabolic Medicine, Level 4, R Wing, Princess Alexandra Hospital, Queensland 4102, Australia; 2 Department of Orthopedics, The Wesley Hospital, Queensland 4066, Australia; 3 Department of Orthopedics, Princess Alexandra Hospital, Queensland 4102, Australia

**Keywords:** osteosarcoma, bone tumour, chemoresistance, RNA expression profile

## Abstract

**Background:**

Osteosarcoma (OS) is the most common primary bone tumour in children and adolescents. Patients who respond poorly to chemotherapy have a higher risk of metastatic disease and 5-year survival rates of only 10–20%. Therefore, identifying molecular targets that are specific for OS, or more specifically, metastatic OS, will be critical to the development of new treatment strategies to improve patient outcomes.

**Methods:**

We performed a transcriptomic analysis of chemo-naive OS biopsies and non-malignant bone biopsies to identify differentially expressed genes specific to OS, which could provide insight into OS biology and chemoresistance.

**Results:**

Statistical analysis of the OS transcriptomes found differential expression of several metallothionein family members, as well as deregulation of genes involved in antigen presentation. Tumours also exhibited significantly increased expression of ID1 and profound down-regulation of S100A8, highlighting their potential as therapeutic targets for OS. Finally, we found a significant correlation between OS and impaired osteoclastogenesis and antigen-presenting activity. The reduced osteoclastogenesis and antigen-presenting activity were more profound in the chemoresistant OS samples.

**Conclusion:**

Our results indicate that OS displays gene signatures consistent with decreased antigen-presenting activity, enhanced chemoresistance, and impaired osteoclastogenesis. Moreover, these alterations are more pronounced in chemoresistant OS tumour samples.

Osteosarcoma (OS) is the most common primary malignant bone tumour in children and adolescents (Chou and Gorlick, 2006). Disease progression is marked by aggressive growth, local recurrence, and poor long-term survival rates because of the development of fatal pulmonary metastasis in up to 50% of patients (Wang, 2005). Chemotherapy over many weeks results in disease-free survival rates of 50–70% for patients with non-metastatic disease (Bacci *et al*, 1997; Marina *et al*, 2004). However, patients who have a poor response to chemotherapy have a higher risk of developing pulmonary metastases, which results in survival rates of <20% (Chou and Gorlick, 2006). To develop new-targeted therapies to treat OS will require knowledge of the specific defects associated with non-metastatic and metastatic disease. Tests to predict whether a patient will respond to current chemotherapeutics or develop metastases would enhance our ability to select appropriate treatment strategies. To this end, a number of studies of OS have generated gene expression signatures, which have provided insights into OS biology (Leonard *et al*, 2003; Baird *et al*, 2005) and chemoresistance (Ochi *et al*, 2004; Man *et al*, 2005; Mintz *et al*, 2005). However, these studies have focused on comparisons of chemosensitive *vs* chemoresistant, or metastatic *vs* non-metastatic disease. Studies comparing non-malignant bone *vs* OS tissue have not been earlier reported.

In this study, we compared the transcriptomes of chemo-naive OS biopsies, collected at the time of diagnosis, with samples of non-malignant bone. Statistical analysis of the expression profiles shows that osteosarcomas are characterised by an early deregulation of genes involved in drug resistance, tumour progression, antigen presentation, and osteoclastogenesis. Furthermore, in biopsies from patients who developed metastatic disease, these changes were significantly more pronounced. These data suggest that patient prognosis is determined early in tumour development and that enhancing antigen presentation or osteoclastogenesis may be of clinical value in treating OS.

## Materials and Methods

### Patient samples

Patients presented to the Oncology Clinic at the Princess Alexandra or at The Wesley Hospitals (Brisbane, Queensland, Australia). Tumour biopsies were collected at the time of initial diagnosis, before preoperative chemotherapy, with informed consent from patients/guardians and with approval from the relevant institutional Research Ethics Committees. Twenty-three biopsies were available and subjected to gene expression profiling analysis. Clinical data detailing response to chemotherapy was available for 22 out of 23 patients (Table 1). Patients were classified as good responders (R) if the tumours had ⩾90% tumour necrosis, or poor responders (N) if the tumours had <90% necrosis in response to preoperative chemotherapy (doxorubicin, 25 mg m^–2^ and cisplatin, 100 mg m^–2^) as determined by histologic examination at the time of definitive surgery (Salzer-Kuntschik *et al*, 1983). Non-malignant bone was collected with consent from five patients presenting for hip or knee replacement surgery.

### Microarray and data analysis

Extraction of RNA from cells and tumours was performed using TRIzol reagent (Invitrogen, Carlsbad, CA, USA). Each OS patient sample was analysed in duplicate using dye swapping. Five non-malignant bone samples were analysed individually. Labelled reference RNA and labelled tumour RNA were combined before hybridisation to Agilent Whole Human Genome Oligo Microarrays (Agilent Technologies, Santa Clara, CA, USA). Arrays were scanned on an Agilent DNA Microarray Scanner G2505B (Agilent Technologies). The microarray data discussed in this work have been deposited in NCBI's Gene Expression Omnibus (GEO; http://www.ncbi.nlm.nih.gov/geo/) and are accessible through GEO series accession number GSE19276. Data extraction was performed using ImaGene version 6.1 (BioDiscovery Inc, El Segundo, CA, USA). Statistical analysis of the data was performed with GeneSpring GX software versions 7.2 and 10.0.1 (Agilent Technologies). Details of the analysis are described in Supplementary Material.

### PCR analysis

For validation, 11 genes were selected at random from the 20 most highly differentially expressed molecules between non-malignant bone and OS. For PCR, 2 *μ*g of non-malignant bone or OS tumour biopsy RNA was reverse transcribed with BioScript (Bioline, Sydney, Australia); PCR was performed using *Taq* DNA polymerase with ThermoPol II buffer (New England Biolabs, Ipswich, MA, USA) at an annealing temperature of 53–55°C for 30 cycles on a ThermoHybaid PxE0.2 (Thermo Scientific, Waltham, MA, USA). Primers were as follows: ID1 (forward 5′-CGGATCTGAGGGAGAACAAG-3′ and reverse 5′-CTGAGAAGCACCAAACGTGA-3′), PRDX4 (forward 5′-GAGGACTTGGGCCAATAAGG-3′ and reverse 5′-TTCACTACCAGGTTTCCAGC-3′), TPM2 (forward 5′-CGAGAGTAAATGTGGGGACC-3′ and reverse 5′-TAAAGGATGAAGCCAGTGCC-3′), MT1E (forward 5′-TGCTTGTTCGTCTCACTGG-3′ and reverse 5′-AAAGAAATGCAGCAAATGGC-3′), FKBP9 (forward 5′-TACCTGAAAACTGTGAGCGG-3′ and reverse 5′-GTTCATCTGGTTTGGCTTCC-3′), S100A13 (forward 5′-ACCTTATGACCTGTCAGCCC-3′ and reverse 5′-CCGAGTCCTGATTCACATCC-3′), S100A8 (forward 5′-TGGGCATCATGTTGACCGAGCTG-3′ and reverse 5′-GCCACGCCCATCTTTATCACCAGA-3′), CTSG (forward 5′-CGCATCTTCGGTTCCTACG-3′ and reverse 5′-GCTTCTCATTGTTGTCCTTATCC-3′), VWA5B2 (forward 5′-TACTCGGGAGCTACTCTTCC-3′ and reverse 5′-CATATGGCTGTGTCAGAGGG-3′), AZU1 (forward 5′-AGCATCAGGTCGTTCAGGTT-3′ and reverse 5′-CAGAATCAAGGCAGGCACTTC-3′), PFC (forward 5′-GCTCTGTCACCTGCTCCAA-3′ and reverse 5′-GCGGCTTCGTGTCTCCTTA-3′).

## Results

### Gene expression profiling of OS *vs* non-malignant bone

**Figure 1:**
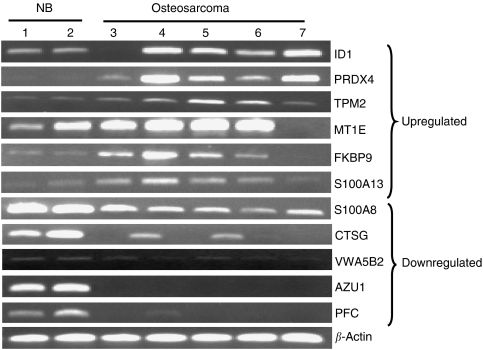
Validation of highly differentially expressed genes in osteosarcoma *vs* non-malignant bone. Eleven genes were selected at random from Table 1 and validated by PCR in two non-malignant bone samples (lanes 1 and 2), and five randomly selected osteosarcoma patients (lanes 3–7). The results are shown in groups of genes upregulated and downregulated in osteosarcoma compared with non-malignant bone.

We compared gene expression in 23 OS biopsies and 5 non-malignant bone samples. Our analysis yielded a suite of 305 differentially expressed genes (two-fold or greater, *P*<0.05) between OS and non-malignant bone, of which 206 were annotated (Supplementary Table 1). Table 2 lists the 10 most highly up and 10 most highly downregulated genes, 11 of which were selected at random and their differential expression confirmed by PCR in two non-malignant bone samples and in five randomly selected OS tumour biopsies (Figure 1). Of the 36 upregulated genes, 47% were associated with cellular growth and proliferation (e.g. ID1, ANXA2, BTG3, MT2A, ITGB1, NDUFAF2). The most well-represented family of genes within this group was the metallothionein family, linked to intrinsic and acquired drug resistance (Cherian *et al*, 2003). Seven members of this family, MT1E, MT1H, MT1X, MT2A, MT1B, MT1G, and MT1L, were upregulated in our OS samples, and three were among the 10 most highly upregulated genes (Table 2). The inhibitor of DNA binding 1, ID1 (+4.07, *P*=0.003), peroxiredoxin 4, PRDX4 (+3.63, *P*=0.007), S100 calcium-binding protein A13, S100A13 (+2.66, *P*=0.009), annexin 2, ANXA2 (+2.62, *P*=0.003), and destrin, DSTN (+2.50, *P*=0.001), earlier reported as positive regulators of angiogenesis, tumour progression and invasion (Fong *et al*, 2003; Landriscina *et al*, 2006; Estornes *et al*, 2007; Lee *et al*, 2008; Mussunoor and Murray, 2008; Iwatsuki *et al*, 2009) were all induced in OS samples (Table 2).

Analysis of the 170 downregulated genes found a large number associated with the inflammatory (26%) and cell-mediated (31%) immune response, as well as with antigen presentation (24%). Ingenuity pathway analysis (IPA) (Ingenuity Systems, Mountain View, California, USA) identified the antigen-presentation pathway as downregulated in OS with HLA-C, HLA-DOA, HLA-DPB1, HLA-DPA1, and HLA-E all expressed 2.17- to 3.45-fold lower in the lesions than in non-malignant bone (*P*=0.002–0.04). However, the most highly differentially expressed gene was the S100 calcium-binding protein A8 (S100A8), a marker of a number of inflammatory conditions (Zreiqat *et al*, 2007), which was downregulated 100-fold in OS (*P*=0.005) (Table 2). Also downregulated were cathepsin G (CTSG; −16.67, *P*=0.02) and azurocidin 1 (AZU1; −6.67, *P*=0.05), which regulate monocyte/macrophage function and chemotaxis in inflammatory conditions (Pereira, 1995; Miyata *et al*, 2007).

**Figure 2:**
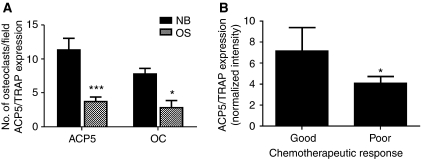
Impaired osteoclastogenesis in osteosarcoma correlates with chemoresistance. (**A**) Osteoclast number is expressed as the average number of osteoclasts per × 20 field in immunohistochemically stained FFPE sections from osteosarcoma biopsies (OS) and non-malignant bone (NB). The ACP5 expression as measured by microarray analysis and expressed as normalised intensity. ^***^P<0.0001, ^*^P=0.0491. Bars: mean+s.e.m. (**B**) ACP5 expression in good and poor responders, as measured by microarray analysis and expressed as normalised intensity. ^*^P=0.0428. Bars: mean+s.e.m.

The OS biopsies also revealed a transcriptomic signature characteristic of reduced osteoclastogenesis. ID1 is an inhibitor of osteoclast differentiation (Lee *et al*, 2006) and was induced four-fold in OS biopsies. Similarly, there was a 100-fold down-regulation of S100A8, which is highly expressed in osteoclasts (Zreiqat *et al*, 2007), and significantly lower expression of another 13 genes associated with negative regulation of osteoclast differentiation/function, or indicating diminished osteoclast presence or activity. These genes included von Willebrand factor A domain, 5B2 (VWA5B2; −11.11, *P*=0.02), FGR, a member of the Src family of protein tyrosine kinases (−5.56, *P*=0.02), TYRO protein kinase-binding protein (TYROBP; −5.26, *P*=0.04), the Rac small GTPase RAC2 (−4.76, *P*=0.01), RelA/p65 (−4.55, *P*=0.005), MYC (−3.70, *P*=2.77 × 10^−16^), signal regulatory protein *α* (SIRP*α*/SIRPA; −2.94, *P*=0.006), tartrate-resistant acid phosphatase (ACP5/TRAP, −2.86, *P*=0.005), BCL2 (−2.5-fold, *P*=0.005), high-mobility group box 1 (HMGB1; −2.33, *P*=0.002), V-ATPase (ATP6V0D1; −2.27, *P*=0.04), leukotriene B4 receptor (LTB4R; −2.22, *P*=0.03), and gelsolin (GSN, −2.08, *P*=0.04). To see whether the reduction in ACP5/TRAP gene expression correlated with a decrease in the number of osteoclasts in OS, we performed immunohistochemistry on FFPE sections of OS biopsies and of non-malignant bone with a monoclonal antibody to ACP5/TRAP. We found a 2.5-fold decrease in the number of osteoclasts in OS biopsies compared with non-malignant bone, which correlated with the observed 2.3-fold decrease in ACP5/TRAP gene expression (Figure 2A). Furthermore, the decrease in ACP5/TRAP expression was significantly more marked in the biopsies of patients, which showed a poor response to chemotherapy treatment than in those who exhibited a good response (Figure 2B), suggesting that a reduction in osteoclastogenesis is not only associated with OS in general, but also with chemoresistance.

### Gene expression profiling of good responders *vs* poor responders

Osteosarcomas are inherently drug-resistant tumours (Chou and Gorlick, 2006), and, therefore, the most commonly used predictor of disease outcome is a patient's initial response to chemotherapy. Unfortunately, this response cannot be assessed at the time of presentation. To specifically search for genes that could be predictive of chemotherapeutic response and drug resistance at the time of diagnosis, patients were divided into good (*n*=5) and poor (*n*=17) responders using the criteria already described. A set of 123 genes was found to be significantly differentially expressed (*P*<0.05) between the two groups, of which 61 were annotated (Table 3). Most of these genes (94%) were upregulated in the good responders and were associated with cellular development, growth, and proliferation, suggesting that good responders may have tumours that are more proliferative and may, therefore, be more sensitive to the effects of chemotherapy. Among the most highly upregulated genes in good responders was thymosin *β* 10 (TSMB10, +5.34-fold, *P*=0.017), which had been identified within the suite of differentially expressed genes between good and poor responders in an earlier study of OS tumour biopsies (Ochi *et al*, 2004).

**Figure 3:**
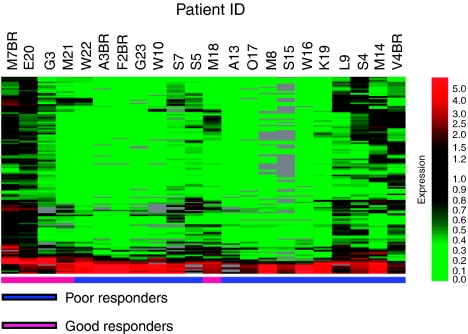
Unsupervised clustering of 123 differentially expressed genes between good responders and poor responders. Genes were selected based on normalised data values that were greater or less in 5 good responders than those in 17 poor responders by a factor of two-fold, with statistically significant differences when grouped by ‘response’ according to a parametric test Welch *t*-test (*P*-value cutoff 0.05). Multiple testing correction was applied (Benjamini and Hochberg false discovery rate). Level of expression – lowest (*light green*), highest (*bright red*).

**Figure 4:**
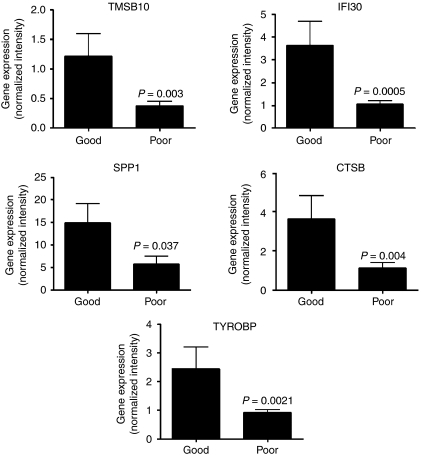
Expression of highly differentially expressed genes between good and poor responders. Expression, measured as normalised spot intensity, in each of the 5 good responders and 17 poor responders of 6 highly differentially expressed genes between the two groups. *P*-value (ANOVA, Welch *T*-test, Benjamini and Hochberg false discovery rate). Bars: s.e.m.

To test the suitability of this set of 123 genes to separate between good and poor responders, we performed unsupervised hierarchical clustering of the data. With the exception of one patient (M18), the gene set was able to clearly separate 21 out of 22 (>95%) OS patients on the basis of their response to chemotherapy (Figure 3). We examined the possibility that some of these genes could serve as individual predictors of chemotherapeutic response. We selected individual genes from the list based on their ⩾three-fold expression (Table 3), and looked at their levels of expression in individual patients. Of the selected genes, only TMSB10, SPP1, CTSB, TYROBP/DAP12, and IFI30 showed significant (*P*<0.05) differential expression between patients in the two groups, with IFI30 (*P*=0.0005) showing the most significant difference (Figure 4).

**Figure 5:**
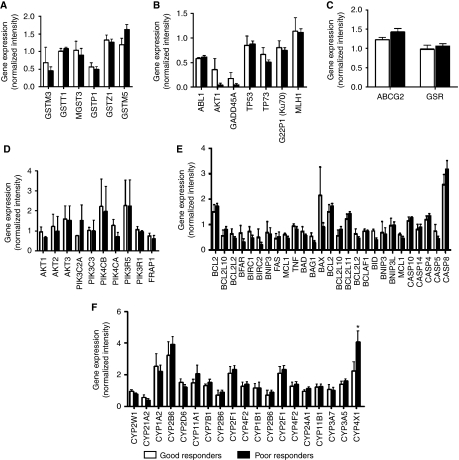
Expression of genes classically associated with chemotherapeutic response. (**A**) Glutathione *S*-transferase family, (**B**) DNA damage response, (**C**) drug transport, (**D**) survival, (**E**) apoptosis, and (**F**) CYP family. ^*^P<0.05. Bars: mean+s.e.m.

One of the major obstacles to effective treatment of OS patients is intrinsic or acquired resistance to the cytotoxic effects of anticancer agents. The mechanism dictating this resistance in OS is still unknown, but may involve a number of gene families, which mediate detoxification, increased efflux from the cell, and increased DNA repair (Chou and Gorlick, 2006). Therefore, understanding the mechanism of drug resistance and identifying genes that are involved may lead to new therapies that could improve survival. The cytochrome P450 family of enzymes functions in the detoxification of anticancer drugs (Simpson, 1997). As our signature found CYP4X1 differentially expressed between good and poor responders, we looked at the mRNA expression levels in each patient of CYP4X1 and other cytochrome P450 family members (Figure 5F). Of the 50 or more P450 genes present on the array, only 19 had detectable expression levels in our tumour biopsies, and of these, only CYP4X1 had significant differential expression associated with chemotherapeutic response. However, given that CYP4X1 is an orphan P450 protein with no assigned biological function (Stark *et al*, 2008), and that it was downregulated in good responders, its function in OS drug response remains unclear.

Other enzyme families responsible for resistance to many chemotherapeutic agents include the glutathione-*S*-transferases (GSTs) (Ekhart *et al*, 2009) and the ATP-binding cassette (ABC) transporters (Sharom, 2008). We, therefore, compared the expression of GST and ABC family members between good and poor responders. Only five GSTs were expressed in our samples, but none significantly (Figure 5A). Similarly, only one ABC transporter, ABCG2, was found expressed in our samples, but its levels were not significantly different between the two groups (Figure 5C). Moreover, we found no significant difference in the expression of other genes involved in DNA damage response, drug metabolism, apoptosis, or survival (Figures 5B, D and E). Taken together, our data indicate that genes classically associated with multi-drug resistance do not correlate with chemotherapeutic response in OS.

## Discussion

Despite intensive multi-agent chemotherapy, OS remains an aggressive, highly metastatic, and relatively drug-resistant tumour with poor long-term survival rates (Chou and Gorlick, 2006). The mechanisms behind metastasis and chemoresistance in OS are not well understood, but are likely to be due to the innate biology of metastatic and chemoresistant lesions (Gorlick and Meyers, 2003). Therefore, understanding the basic tumour biology is central to understanding OS pathogenesis and chemoresistance. In this study, we used chemo-naive OS biopsies and, for the first time, compared their transcriptomes to those of non-malignant bone. We identified a unique gene signature showing increased expression of genes associated with tumour progression and drug resistance, and decreased expression of genes associated with antigen presentation and osteoclastogenesis in all OS lesions. In addition, tumours that were chemoresistant were characterised by more pronounced inhibition of osteoclastogenesis markers and antigen-presenting activity than tumours that were chemosensitive. We noted no significant difference in the expression of genes classically associated with drug resistance between chemosensitive and chemoresistant tumours.

This study identified ID1 as a potentially important molecule in the regulation of many of the characteristics of OS. Increased expression of ID1 in the OS biopsies has been shown to be involved in the proliferation, survival, angiogenesis, metastasis (Ling *et al*, 2006), and formation of a permissive metastatic niche in other cancer types (Lyden *et al*, 1999), and may be similarly involved in OS. Recently, ID1 has been identified as a novel inhibitor of PTEN and p53, leading to AKT-dependent activation of the canonical Wnt-signalling pathway (Lee *et al*, 2009). The Wnt-signalling pathway has earlier been shown to be important to the regulation of OS progression (Hoang *et al*, 2004; Geryk-Hall and Hughes, 2009; Kansara *et al*, 2009). Thus, there is evidence that the overexpression of ID1 in OS may be causally involved in the growth, survival, and metastatic behaviour of OS. If true, then targeting of the Akt and Wnt pathways could be of value in OS (Geryk-Hall and Hughes, 2009).

Mintz *et al* (2005) earlier reported that genes involved in osteoclast differentiation and function in OS were mostly associated with a poor chemotherapeutic response. We now report that osteoclast numbers are decreased in all OS lesions. Thus, the loss of osteoclasts in OS may be involved in OS metastasis, although the mechanism by which OS induce osteoclast loss is unknown. An earlier transcriptomic study in seven OS biopsies (Patino-Garcia *et al*, 2009) showed up-regulation of EBF2, a known transcription factor for osteoprotegerin (OPG), a major negative regulator of osteoclastogenesis (Khosla, 2001). In contrast, our study identified the up-regulation of ID1 in OS. As ID1 is a negative regulator of osteoclast differentiation (Lee *et al*, 2006), it is possible that the overexpression of ID1 in OS lesions may provide an explanation for OS-induced osteoclast loss. Our microarray analysis of primary OS lesions also indicates that S100A8 was profoundly reduced in OS lesions (*P*=0.005; fold decrease=100). S100A8 is highly expressed in osteoclasts in which it functions as a chemotactic-signalling molecule involved in the coupling of osteoclast and osteoblast activity (Zreiqat *et al*, 2007). In addition, decreased expression of 15 other genes involved in osteoclast development and function (Pereira *et al*, 1990; Garcia *et al*, 1996; Lowell *et al*, 1996; Chertov *et al*, 1997; Chellaiah *et al*, 2000; Duong *et al*, 2000; Battaglino *et al*, 2002; Lundberg *et al*, 2007; Shahbazi *et al*, 2007; Kawano *et al*, 2008; Zhou *et al*, 2008; Soehnlein and Lindbom, 2009) was observed in the OS biopsies. Among these was CTSG, which is necessary for the recruitment of osteoclast precursors (Wilson *et al*, 2009b) and for the activation of MMP9, which in turn activates TGF*β* to enhance osteoclast activity (Wilson *et al*, 2009a); TYROBP/DAP12, which is essential for RANK signalling and osteoclast multinucleation and differentiation (Humphrey *et al*, 2004; Mocsai *et al*, 2004), and the NFkB subunit, RelA/p65, which promotes osteoclastogenesis by inhibiting JNK-mediated osteoclast apoptosis (Vaira *et al*, 2008; Soysa and Alles, 2009). We also observed decreased expression of osteoclast cellular components such as ACP5/TRAP, a classic marker of mature and active osteoclasts (Hayman, 2008) and ATP6V0D1, which is found in the osteoclast membrane and critical for its resorptive activity (Xu *et al*, 2007). Furthermore, the decreased expression of osteoclast cellular components correlated with a decrease in the number of osteoclast cell counts in immunohistochemically stained sections of OS compared with non-malignant bone. Thus, we provide evidence that OS lesions are associated with a reduction in osteoclasts.

The down-regulation of osteoclast differentiation in OS was accompanied by down-regulation of five MHC Class I and II genes belonging to the antigen-presentation pathway and of HMGB1. The correlation between MHC Class I and II deficiencies in human tumours and metastatic potential/reduced survival rates in patients is well documented (Meissner *et al*, 2005; Chamuleau *et al*, 2006; Ramnath *et al*, 2006; Seliger, 2008), whereas the interaction of HMGB1 with Toll-like receptor 4 on dendritic cells has been shown to be essential for tumour antigen processing and presentation (Apetoh *et al*, 2007). Interestingly, we observed a significant increase in the expression of five genes with overlapping functions in antigen presentation and osteoclastogenesis in the good responder group – IFI30 (*P*=0.0005), TYROBP/DAP12 (*P*=0.002), TMSB10 (*P*=0.003), CTSB (*P*=0.004), and SPP1 (*P*=0.037). The IFI30 expression, in melanoma (Goldstein *et al*, 2008) and squamous cell carcinoma (Wenzel *et al*, 2008), has been shown to enhance antigen presentation and to activate CTSB (Goldstein *et al*, 2008), which is required for osteoclast fusion during differentiation (McMichael *et al*, 2009). In addition, TYROBP/DAP12 is associated with active innate immune responses (Lanier, 2009) and, together with SPP1, also has a crucial function in osteoclast differentiation (Humphrey *et al*, 2004; Mocsai *et al*, 2004; Dalla-Torre *et al*, 2006; Inui *et al*, 2009). Furthermore, the expression of SPP1 in OS lesions has been correlated with improved overall survival (Dalla-Torre *et al*, 2006). Although there is some evidence connecting TMSB10 expression to carcinogenesis (Lee *et al*, 2001; Sardi *et al*, 2002; Alldinger *et al*, 2005), this is the first report linking TMSB10 expression to good chemotherapeutic response in OS. In addition, good responders had significantly higher levels of ACP5/TRAP gene expression than poor responders. Thus, these results are the first to show a possible association between antigen presentation and osteoclastogenesis in the biology of OS tumours and in their chemotherapeutic response.

This study revealed two interesting findings relating to OS chemosensitivity. First, we found that OS displayed a gene signature that was consistent with a chemoresistant phenotype. Second, genes associated with classical drug resistance were not overrepresented in chemoresistant OS lesions. These conclusions are supported by the increased expression of seven metallothionein family members in the OS biopsies. The function of metallothioneins in drug resistance has been well documented (Cherian *et al*, 2003; Theocharis *et al*, 2004; Surowiak *et al*, 2007), and an earlier transcriptomic study of OS reported up-regulation of MTIG and MT1L in poorly responsive tumours (Mintz *et al*, 2005). In this study, we found no correlation between their level of expression and response, a finding that is supported by others (Uozaki *et al*, 1997; Shnyder *et al*, 1998). Moreover, we found no correlation between good and poor responses and the expression of molecules classically associated with chemoresistance, such as GSTs and ABC transporters, or DNA damage response, apoptosis, drug metabolism, and survival genes. These results suggest that drug resistance may be a global characteristic of all osteosarcomas and that drug resistance, in chemoresistant lesions, is likely to be mediated by novel pathways.

## Figures and Tables

**Table 1:** Clinical information for the osteosarcoma patients used in the study

**Tumour ID**	**Age (years)**	**Gender**	**Site of tumour**	**Tumour necrosis (%)**	**Response**
G3	15	M	Proximal tibia	100	R
S4	29	F	Sacrum	<5	N
S5	27	F	Proximal humerus	<50	N
S7	39	M	Pelvis	<50	N
M8	24	F	Distal femur	25	N
L9	17	M	Tibia	75	N
W10	14	M	Distal femur	75	N
A13	7	F	Proximal tibia	80	N
M14	67	M	Pelvis	75	N
S15	76	F	Tibia	<90	N
W16	15	F	Proximal Humerus	<90	N
O17	12	M	Distal femur	75	N
M18	18	M	8th rib	98	R
K19	17	M	Proximal tibia	50	N
E20	13	F	Femur	95	R
M21	15	M	Distal femur	95	R
W22	15	F	Femur	80	N
G23	18	M	Distal femur	80	N
F2BR	18	M	Proximal tibia	80	N
A3BR	19	F	Distal femur	80	N
V4BR	16	M	Calcaneum	<5	N
M7BR	14	F	Distal femur	>92	R
T1^a^	37	F	Proximal tibia	U	U

Response to chemotherapy: R=good response; N=poor response; U=unknown.

a This patient was used in the comparison between osteosarcoma and non-malignant bone, but not in the chemotherapy response study.

**Table 2:** Osteosarcoma *vs* non-malignant bone

**Probe ID**	**Symbol**	**Description and accession number**	**Fold change**	***P*-value**
A_23_P252306	ID1	Inhibitor of DNA binding 1, dominant-negative helix-loop-helix protein [NM_002165]	4.07	0.00276
A_23_P114232	PRDX4	Peroxiredoxin 4 [NM_006406]	3.63	0.00664
A_23_P216501	TPM2	Tropomyosin 2 (*β*) [NM_213674]	3.30	0.00237
A_23_P206724	MT1E	Metallothionein 1E [NM_175617]	3.11	0.00661
A_23_P334709	FKBP9	FK506-binding protein 9, 63 kDa [NM_007270]	3.08	0.0111
A_23_P163782	MT1H	Metallothionein 1H [NM_005951]	3.02	0.0169
A_24_P125096	MT1X	Metallothionein 1X [NM_005952]	2.72	0.0316
A_23_P372874	S100A13	S100 calcium-binding protein A13 [NM_005979]	2.66	0.00955
A_32_P94798	ANXA2	Annexin A2 [NM_001002857]	2.62	0.00359
A_23_P408095	DSTN	Destrin (actin depolymerising factor) [NM_001011546]	2.50	0.00140
A_23_P434809	S100A8	S100 calcium-binding protein A8 (calgranulin A) [NM_002964]	−100.00	0.00549
A_23_P37856	HBA1	Homo sapiens haemoglobin, *α* 1 (HBA1), mRNA [NM_000558]	−50.00	4.26E−10
A_23_P140384	CTSG	Cathepsin G [NM_001911]	−16.67	0.0222
A_23_P80867	VWA5B2	von Willebrand factor A domain containing 5B2 [AL834499]	−11.11	0.0222
A_23_P153741	AZU1	Azurocidin 1 (cationic antimicrobial protein 37) [NM_001700]	−6.67	0.0485
A_23_P22444	CFP	Properdin P factor, complement [NM_002621]	−6.25	0.0496
A_23_P208866	GMFG	Glia maturation factor, *γ* [NM_004877]	−5.88	0.00748
A_24_P207195	IRX3	Iroquois homeobox protein 3 [NM_024336]	−5.88	0.00263
A_23_P403886	GLYAT	Glycine-*N*-acyltransferase [NM_005838]	−5.56	0.00391
A_23_P156708	TNXB	Tenascin XB [NM_019105]	−5.56	0.00351

Top 10 upregulated and 10 downregulated genes between osteosarcoma biopsies and non-malignant bone samples.

**Table 3:** Good response *vs* poor response

**Genbank**	**Synonym**	**Common name**	**Fold change**	***P*-value**
NM_021103	TMSB10	Thymosin, *β* 10	5.34	0.0171
NM_000582	SPP1	Secreted phosphoprotein 1 (osteopontin, bone sialoprotein I, early T-lymphocyte activation 1)	4.82	0.0171
NM_006332	IFI30	Interferon, *γ*-inducible protein 30	4.36	0.0224
NM_002952	RPS2	Ribosomal protein S2	3.70	0.0487
NM_005514	HLA-B	Major histocompatibility complex, class I, B	3.56	0.0487
NM_000455	STK11	Serine/threonine kinase 11 (Peutz–Jeghers syndrome)	3.49	0.0249
NM_147780	CTSB	Cathepsin B	3.48	0.0355
NM_153461	IL17RC	Interleukin 17 receptor C	3.39	0.0415
NM_002032	FTH1	Ferritin, heavy polypeptide 1	3.32	0.0355
NM_005507	CFL1	Cofilin 1 (non-muscle)	3.29	0.0251
NM_002116	HLA-A	Major histocompatibility complex, class I, A	3.17	0.0379
NM_003332	TYROBP	TYRO protein tyrosine kinase-binding protein	3.16	0.0487
NM_001022	RPS19	Ribosomal protein S19	3.16	0.023
NM_053275	RPLP0	Ribosomal protein, large, P0	3.14	0.0439
NM_000992	RPL29	Ribosomal protein L29 a.k.a. heparin/eparan sulphate interacting protein (HIP)	3.11	0.0497
NM_002117	HLA-C	Major histocompatibility complex, class I, C	2.99	0.0401
NR_002205	FTHL12	Ferritin, heavy polypeptide-like 12	2.95	0.0392
NM_001404	EEF1G	Eukaryotic translation elongation factor 1 *γ*	2.93	0.0443
NM_001014	RPS10	Ribosomal protein S10	2.83	0.0171
NM_001003	RPLP1	Ribosomal protein, large, P1	2.81	0.0397
NM_003295	TPT1	Tumour protein, translationally controlled 1	2.66	0.0365
BC031631	CFLP1	Cofilin pseudogene 1	2.65	0.0232
NM_006435	IFITM2	Interferon induced transmembrane protein 2 (1-8D)	2.61	0.0489
NM_002948	RPL15	Ribosomal protein L15	2.60	0.0497
NM_001020	RPS16	Ribosomal protein S16	2.58	0.0355
NM_000982	RPL21	Ribosomal protein L21	2.56	0.0489
NM_178230	COAS2	Cyclophilin-LC	2.51	0.0444
NM_000979	RPL18	Ribosomal protein L18	2.50	0.0455
NM_001022	RPS19	Ribosomal protein S19	2.47	0.0392
NM_003295	TPT1	Tumour protein, translationally controlled 1	2.47	0.0439
BC034271	FANCC	Fanconi anaemia, complementation group C	2.45	0.0214
NM_001997	FAU	Finkel–Biskis–Reilly murine sarcoma virus (FBR-MuSV) ubiquitously expressed (fox derived); RPS30	2.45	0.043
NM_001006	RPS3A	Ribosomal protein S3A	2.45	0.0497
AY358369	SIGLEC5	Sialic acid-binding Ig-like lectin 5 (CD170)	2.45	0.0489
NM_181468	ITGB4BP	Integrin *β* 4-binding protein	2.40	0.0444
NM_000990	RPL27A	Ribosomal protein L27a	2.39	0.0392
NM_002489	NDUFA4	NADH dehydrogenase (ubiquinone) 1 *α* subcomplex, 4, 9 kDa	2.32	0.0439
AK098605	FMN2	Formin 2	2.32	0.0443
NM_001019	RPS15A	Ribosomal protein S15a	2.32	0.0401
NM_002107	H3F3A	H3 histone, family 3A	2.30	0.0303
NM_005620	S100A11	S100 calcium-binding protein A11 (calgizzarin)	2.30	0.0487
NM_006013	RPL10	Ribosomal protein L10	2.28	0.0489
NM_005009	NME4	Non-metastatic cells 4, protein expressed in	2.27	0.0365
NM_006886	ATP5E	ATP synthase, H+ transporting, mitochondrial F1 complex, *å* subunit	2.23	0.0171
NM_001008741	LOC388817	Peptidylprolyl isomerase A-like	2.23	0.0444
NM_032828	ZNF587	Zinc-finger protein 587	2.22	0.0487
NM_015933	HSPC016	Hypothetical protein HSPC016	2.22	0.0258
NM_024040	CUEDC2	CUE domain containing 2	2.19	0.0214
NM_006808	SEC61B	Sec61 *β* subunit	2.18	0.0465
NM_002406	MGAT1	Mannosyl (*α*-1,3-)-glycoprotein *β*-1,2-N-acetylglucosaminyltransferase	2.14	0.0415
NM_002797	PSMB5	Proteasome (prosome, macropain) subunit, *β* type, 5	2.13	0.0171
NM_001021	RPS17	Ribosomal protein S17	2.10	0.0489
NM_000182	HADHA	Hydroxyacyl-coenzyme A dehydrogenase/3-ketoacyl-coenzyme A thiolase/enoyl-coenzyme A hydratase (trifunctional protein), *α* subunit	2.07	0.0392
NM_012067	AKR7A3	Aldo-keto reductase family 7, member A3 (aflatoxin aldehyde reductase)	2.06	0.0224
XM_376787	RPS26P10	Ribosomal protein S26 pseudogene 10	2.06	0.043
NM_005340	HINT1	Histidine triad nucleotide-binding protein 1	2.04	0.0444
NM_145893	A2BP1	Ataxin 2-binding protein 1	−2.44	0.0357
NM_015503	SH2B1	SH2-B adaptor protein	−2.33	0.0487
NM_178033	CYP4X1	Cytochrome P450, family 4, subfamily X, polypeptide 1	−2.08	0.0487
NM_003893	LDB1	LIM domain-binding 1	−2.04	0.0355
CR749256	XRCC2	X-ray repair complementing defective repair in Chinese hamster cells 2	−2.00	0.0357

Genes differentially expressed between biopsies of good responders and poor responders. The list shows 61 annotated genes from the original list of 123 genes.
