# Supplementary material for: Osteosarcoma is characterised by reduced expression of markers of osteoclastogenesis and antigen presentation compared with normal bone
Source: Br J Cancer. 2010 Jun 15;103(1):73–81. doi: 10.1038/sj.bjc.6605723 (PMC2905286; doi:10.1038/sj.bjc.6605723)
Supplement: Supplementary Table 1 [file 6605723x1.ppt]

## Slide 1
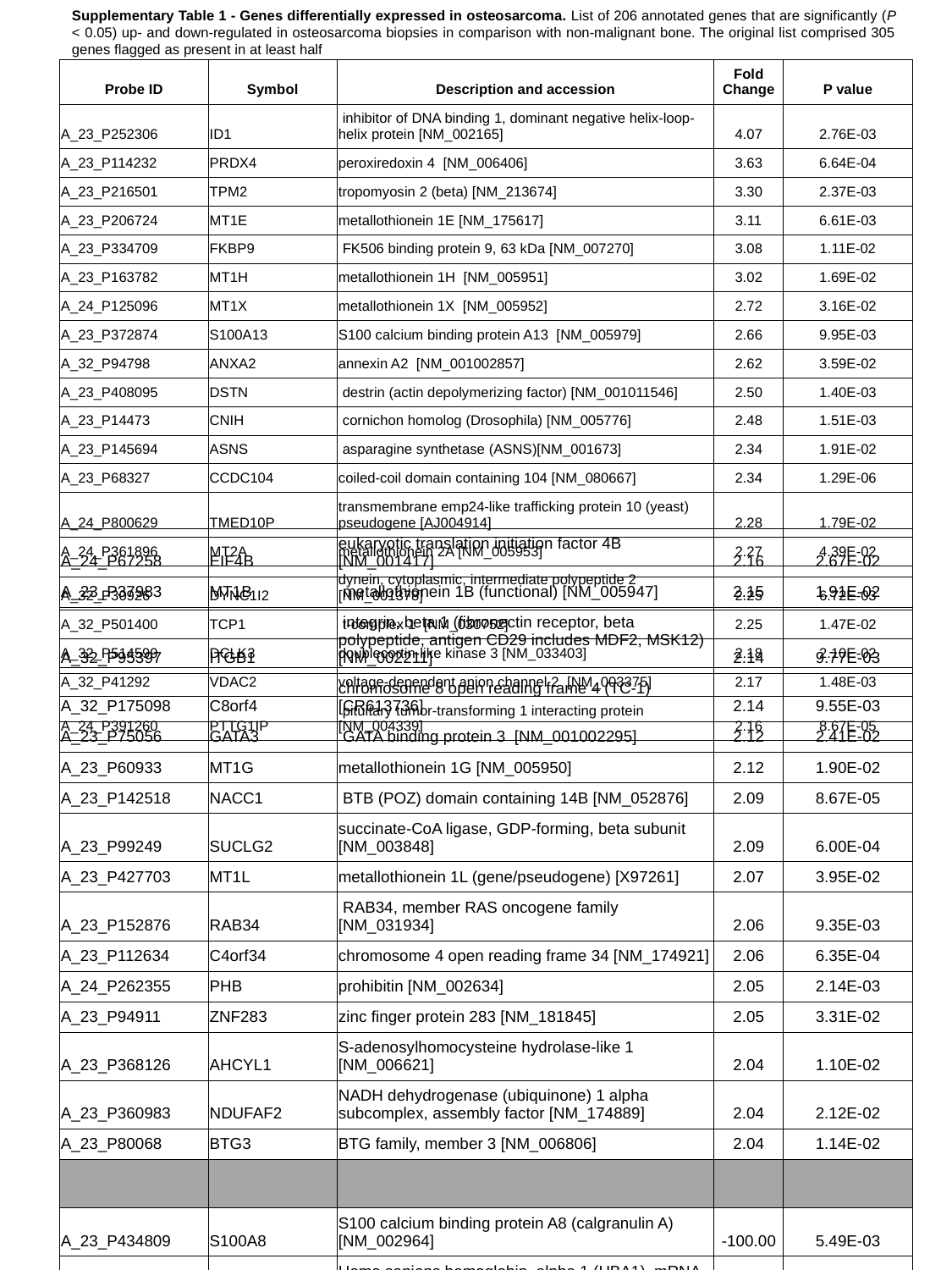

Supplementary Table 1 - Genes differentially expressed in osteosarcoma. List of 206 annotated genes that are significantly (P < 0.05) up- and down-regulated in osteosarcoma biopsies in comparison with non-malignant bone. The original list comprised 305 genes flagged as present in at least half
| Probe ID | Symbol | Description and accession | Fold Change | P value |
| --- | --- | --- | --- | --- |
| A\_23\_P252306 | ID1 | inhibitor of DNA binding 1, dominant negative helix-loop-helix protein [NM\_002165] | 4.07 | 2.76E-03 |
| A\_23\_P114232 | PRDX4 | peroxiredoxin 4 [NM\_006406] | 3.63 | 6.64E-04 |
| A\_23\_P216501 | TPM2 | tropomyosin 2 (beta) [NM\_213674] | 3.30 | 2.37E-03 |
| A\_23\_P206724 | MT1E | metallothionein 1E [NM\_175617] | 3.11 | 6.61E-03 |
| A\_23\_P334709 | FKBP9 | FK506 binding protein 9, 63 kDa [NM\_007270] | 3.08 | 1.11E-02 |
| A\_23\_P163782 | MT1H | metallothionein 1H [NM\_005951] | 3.02 | 1.69E-02 |
| A\_24\_P125096 | MT1X | metallothionein 1X [NM\_005952] | 2.72 | 3.16E-02 |
| A\_23\_P372874 | S100A13 | S100 calcium binding protein A13 [NM\_005979] | 2.66 | 9.95E-03 |
| A\_32\_P94798 | ANXA2 | annexin A2 [NM\_001002857] | 2.62 | 3.59E-02 |
| A\_23\_P408095 | DSTN | destrin (actin depolymerizing factor) [NM\_001011546] | 2.50 | 1.40E-03 |
| A\_23\_P14473 | CNIH | cornichon homolog (Drosophila) [NM\_005776] | 2.48 | 1.51E-03 |
| A\_23\_P145694 | ASNS | asparagine synthetase (ASNS)[NM\_001673] | 2.34 | 1.91E-02 |
| A\_23\_P68327 | CCDC104 | coiled-coil domain containing 104 [NM\_080667] | 2.34 | 1.29E-06 |
| A\_24\_P800629 | TMED10P | transmembrane emp24-like trafficking protein 10 (yeast) pseudogene [AJ004914] | 2.28 | 1.79E-02 |
| A\_24\_P361896 | MT2A | metallothionein 2A [NM\_005953] | 2.27 | 4.39E-02 |
| A\_32\_P30926 | DYNC1I2 | dynein, cytoplasmic, intermediate polypeptide 2 [NM\_001378] | 2.25 | 6.72E-03 |
| A\_32\_P501400 | TCP1 | t-complex 1 [NM\_030752] | 2.25 | 1.47E-02 |
| A\_32\_P514599 | DCLK3 | doublecortin-like kinase 3 [NM\_033403] | 2.18 | 2.10E-02 |
| A\_32\_P41292 | VDAC2 | voltage-dependent anion channel 2 [NM\_003375] | 2.17 | 1.48E-03 |
| A\_24\_P391260 | PTTG1IP | pituitary tumor-transforming 1 interacting protein [NM\_004339] | 2.16 | 8.67E-05 |
| A\_24\_P67258 | EIF4B | eukaryotic translation initiation factor 4B [NM\_001417] | 2.16 | 2.67E-02 |
| --- | --- | --- | --- | --- |
| A\_23\_P37983 | MT1B | metallothionein 1B (functional) [NM\_005947] | 2.15 | 1.91E-02 |
| A\_32\_P95397 | ITGB1 | integrin, beta 1 (fibronectin receptor, beta polypeptide, antigen CD29 includes MDF2, MSK12) [NM\_002211] | 2.14 | 9.77E-03 |
| A\_32\_P175098 | C8orf4 | chromosome 8 open reading frame 4 (TC-1) [CR613736] | 2.14 | 9.55E-03 |
| A\_23\_P75056 | GATA3 | GATA binding protein 3 [NM\_001002295] | 2.12 | 2.41E-02 |
| A\_23\_P60933 | MT1G | metallothionein 1G [NM\_005950] | 2.12 | 1.90E-02 |
| A\_23\_P142518 | NACC1 | BTB (POZ) domain containing 14B [NM\_052876] | 2.09 | 8.67E-05 |
| A\_23\_P99249 | SUCLG2 | succinate-CoA ligase, GDP-forming, beta subunit [NM\_003848] | 2.09 | 6.00E-04 |
| A\_23\_P427703 | MT1L | metallothionein 1L (gene/pseudogene) [X97261] | 2.07 | 3.95E-02 |
| A\_23\_P152876 | RAB34 | RAB34, member RAS oncogene family [NM\_031934] | 2.06 | 9.35E-03 |
| A\_23\_P112634 | C4orf34 | chromosome 4 open reading frame 34 [NM\_174921] | 2.06 | 6.35E-04 |
| A\_24\_P262355 | PHB | prohibitin [NM\_002634] | 2.05 | 2.14E-03 |
| A\_23\_P94911 | ZNF283 | zinc finger protein 283 [NM\_181845] | 2.05 | 3.31E-02 |
| A\_23\_P368126 | AHCYL1 | S-adenosylhomocysteine hydrolase-like 1 [NM\_006621] | 2.04 | 1.10E-02 |
| A\_23\_P360983 | NDUFAF2 | NADH dehydrogenase (ubiquinone) 1 alpha subcomplex, assembly factor [NM\_174889] | 2.04 | 2.12E-02 |
| A\_23\_P80068 | BTG3 | BTG family, member 3 [NM\_006806] | 2.04 | 1.14E-02 |
| | | | | |
| A\_23\_P434809 | S100A8 | S100 calcium binding protein A8 (calgranulin A) [NM\_002964] | -100.00 | 5.49E-03 |
| A\_23\_P37856 | HBA1 | Homo sapiens hemoglobin, alpha 1 (HBA1), mRNA [NM\_000558] | -50.00 | 4.26E-10 |
| A\_23\_P140384 | CTSG | cathepsin G [NM\_001911] | -16.67 | 2.22E-02 |
| A\_23\_P80867 | VWA5B2 | von Willebrand factor A domain containing 5B2 [AL834499] | -11.11 | 2.22E-02 |
| A\_23\_P15055 | HBM | hemoglobin mu chain [NM\_001003938] | -11.11 | 4.44E-02 |
| A\_24\_P831309 | FLJ45717 | Homo sapiens FLJ45717 protein [NM\_207401] | -5.88 | 9.40E-03 |

## Slide 2
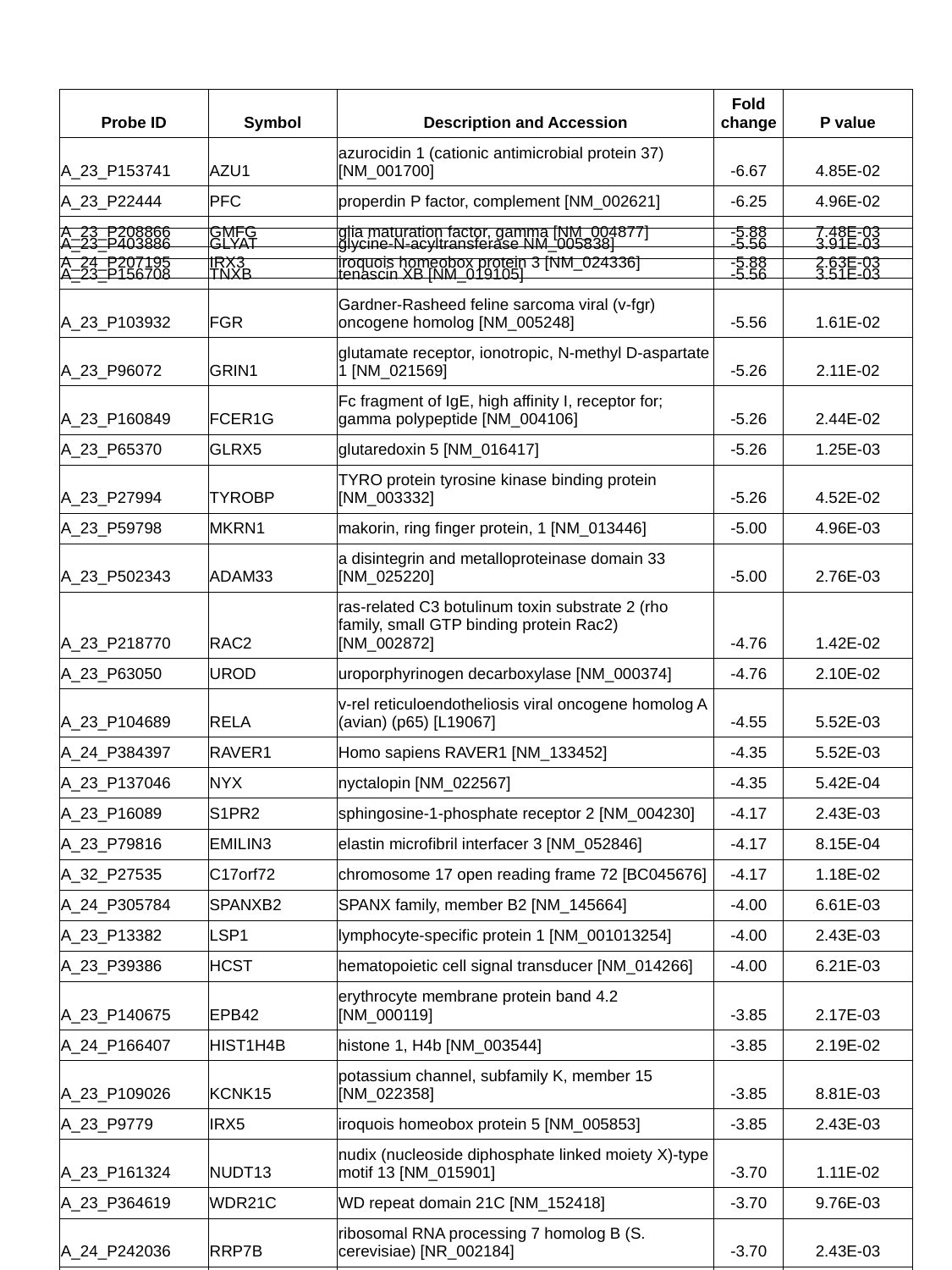

| Probe ID | Symbol | Description and Accession | Fold change | P value |
| --- | --- | --- | --- | --- |
| A\_23\_P153741 | AZU1 | azurocidin 1 (cationic antimicrobial protein 37)[NM\_001700] | -6.67 | 4.85E-02 |
| A\_23\_P22444 | PFC | properdin P factor, complement [NM\_002621] | -6.25 | 4.96E-02 |
| A\_23\_P208866 | GMFG | glia maturation factor, gamma [NM\_004877] | -5.88 | 7.48E-03 |
| A\_24\_P207195 | IRX3 | iroquois homeobox protein 3 [NM\_024336] | -5.88 | 2.63E-03 |
| A\_23\_P403886 | GLYAT | glycine-N-acyltransferase NM\_005838] | -5.56 | 3.91E-03 |
| --- | --- | --- | --- | --- |
| A\_23\_P156708 | TNXB | tenascin XB [NM\_019105] | -5.56 | 3.51E-03 |
| A\_23\_P103932 | FGR | Gardner-Rasheed feline sarcoma viral (v-fgr) oncogene homolog [NM\_005248] | -5.56 | 1.61E-02 |
| A\_23\_P96072 | GRIN1 | glutamate receptor, ionotropic, N-methyl D-aspartate 1 [NM\_021569] | -5.26 | 2.11E-02 |
| A\_23\_P160849 | FCER1G | Fc fragment of IgE, high affinity I, receptor for; gamma polypeptide [NM\_004106] | -5.26 | 2.44E-02 |
| A\_23\_P65370 | GLRX5 | glutaredoxin 5 [NM\_016417] | -5.26 | 1.25E-03 |
| A\_23\_P27994 | TYROBP | TYRO protein tyrosine kinase binding protein [NM\_003332] | -5.26 | 4.52E-02 |
| A\_23\_P59798 | MKRN1 | makorin, ring finger protein, 1 [NM\_013446] | -5.00 | 4.96E-03 |
| A\_23\_P502343 | ADAM33 | a disintegrin and metalloproteinase domain 33 [NM\_025220] | -5.00 | 2.76E-03 |
| A\_23\_P218770 | RAC2 | ras-related C3 botulinum toxin substrate 2 (rho family, small GTP binding protein Rac2) [NM\_002872] | -4.76 | 1.42E-02 |
| A\_23\_P63050 | UROD | uroporphyrinogen decarboxylase [NM\_000374] | -4.76 | 2.10E-02 |
| A\_23\_P104689 | RELA | v-rel reticuloendotheliosis viral oncogene homolog A (avian) (p65) [L19067] | -4.55 | 5.52E-03 |
| A\_24\_P384397 | RAVER1 | Homo sapiens RAVER1 [NM\_133452] | -4.35 | 5.52E-03 |
| A\_23\_P137046 | NYX | nyctalopin [NM\_022567] | -4.35 | 5.42E-04 |
| A\_23\_P16089 | S1PR2 | sphingosine-1-phosphate receptor 2 [NM\_004230] | -4.17 | 2.43E-03 |
| A\_23\_P79816 | EMILIN3 | elastin microfibril interfacer 3 [NM\_052846] | -4.17 | 8.15E-04 |
| A\_32\_P27535 | C17orf72 | chromosome 17 open reading frame 72 [BC045676] | -4.17 | 1.18E-02 |
| A\_24\_P305784 | SPANXB2 | SPANX family, member B2 [NM\_145664] | -4.00 | 6.61E-03 |
| A\_23\_P13382 | LSP1 | lymphocyte-specific protein 1 [NM\_001013254] | -4.00 | 2.43E-03 |
| A\_23\_P39386 | HCST | hematopoietic cell signal transducer [NM\_014266] | -4.00 | 6.21E-03 |
| A\_23\_P140675 | EPB42 | erythrocyte membrane protein band 4.2 [NM\_000119] | -3.85 | 2.17E-03 |
| A\_24\_P166407 | HIST1H4B | histone 1, H4b [NM\_003544] | -3.85 | 2.19E-02 |
| A\_23\_P109026 | KCNK15 | potassium channel, subfamily K, member 15 [NM\_022358] | -3.85 | 8.81E-03 |
| A\_23\_P9779 | IRX5 | iroquois homeobox protein 5 [NM\_005853] | -3.85 | 2.43E-03 |
| A\_23\_P161324 | NUDT13 | nudix (nucleoside diphosphate linked moiety X)-type motif 13 [NM\_015901] | -3.70 | 1.11E-02 |
| A\_23\_P364619 | WDR21C | WD repeat domain 21C [NM\_152418] | -3.70 | 9.76E-03 |
| A\_24\_P242036 | RRP7B | ribosomal RNA processing 7 homolog B (S. cerevisiae) [NR\_002184] | -3.70 | 2.43E-03 |
| A\_24\_P40061 | LENG8 | leukocyte receptor cluster (LRC) member 8 [NM\_052925] | -3.70 | 2.43E-03 |
| A\_24\_P357037 | UBE2G2 | ubiquitin-conjugating enzyme E2G 2 (UBC7 homolog, yeast) [NM\_182688] | -3.57 | 1.95E-03 |
| A\_23\_P27353 | SLC14A2 | solute carrier family 14 (urea transporter), member 2 [NM\_007163] | -3.57 | 2.75E-02 |
| A\_23\_P158829 | ARRB2 | arrestin, beta 2 [NM\_004313] | -3.57 | 4.60E-03 |
| A\_23\_P150162 | DRD4 | dopamine receptor D4 [NM\_000797] | -3.57 | 2.37E-02 |
| A\_23\_P138706 | ADRA2A | adrenergic, alpha-2A-, receptor [NM\_000681] | -3.57 | 1.11E-02 |
| A\_23\_P38795 | FPR1 | formyl peptide receptor 1 [NM\_002029] | -3.57 | 2.08E-02 |

## Slide 3
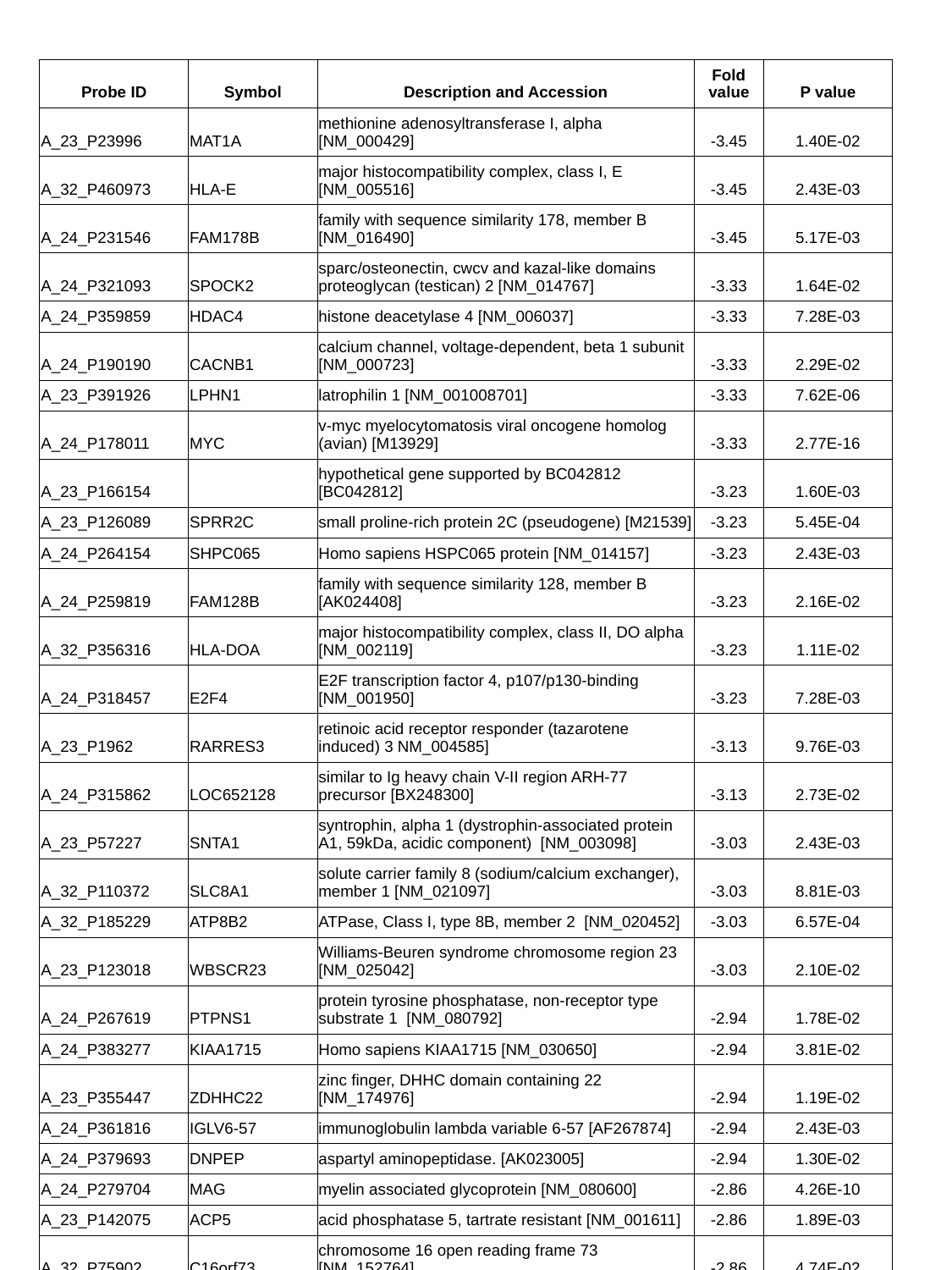

| Probe ID | Symbol | Description and Accession | Fold value | P value |
| --- | --- | --- | --- | --- |
| A\_23\_P23996 | MAT1A | methionine adenosyltransferase I, alpha [NM\_000429] | -3.45 | 1.40E-02 |
| A\_32\_P460973 | HLA-E | major histocompatibility complex, class I, E [NM\_005516] | -3.45 | 2.43E-03 |
| A\_24\_P231546 | FAM178B | family with sequence similarity 178, member B [NM\_016490] | -3.45 | 5.17E-03 |
| A\_24\_P321093 | SPOCK2 | sparc/osteonectin, cwcv and kazal-like domains proteoglycan (testican) 2 [NM\_014767] | -3.33 | 1.64E-02 |
| A\_24\_P359859 | HDAC4 | histone deacetylase 4 [NM\_006037] | -3.33 | 7.28E-03 |
| A\_24\_P190190 | CACNB1 | calcium channel, voltage-dependent, beta 1 subunit [NM\_000723] | -3.33 | 2.29E-02 |
| A\_23\_P391926 | LPHN1 | latrophilin 1 [NM\_001008701] | -3.33 | 7.62E-06 |
| A\_24\_P178011 | MYC | v-myc myelocytomatosis viral oncogene homolog (avian) [M13929] | -3.33 | 2.77E-16 |
| A\_23\_P166154 | | hypothetical gene supported by BC042812 [BC042812] | -3.23 | 1.60E-03 |
| A\_23\_P126089 | SPRR2C | small proline-rich protein 2C (pseudogene) [M21539] | -3.23 | 5.45E-04 |
| A\_24\_P264154 | SHPC065 | Homo sapiens HSPC065 protein [NM\_014157] | -3.23 | 2.43E-03 |
| A\_24\_P259819 | FAM128B | family with sequence similarity 128, member B [AK024408] | -3.23 | 2.16E-02 |
| A\_32\_P356316 | HLA-DOA | major histocompatibility complex, class II, DO alpha [NM\_002119] | -3.23 | 1.11E-02 |
| A\_24\_P318457 | E2F4 | E2F transcription factor 4, p107/p130-binding [NM\_001950] | -3.23 | 7.28E-03 |
| A\_23\_P1962 | RARRES3 | retinoic acid receptor responder (tazarotene induced) 3 NM\_004585] | -3.13 | 9.76E-03 |
| A\_24\_P315862 | LOC652128 | similar to Ig heavy chain V-II region ARH-77 precursor [BX248300] | -3.13 | 2.73E-02 |
| A\_23\_P57227 | SNTA1 | syntrophin, alpha 1 (dystrophin-associated protein A1, 59kDa, acidic component) [NM\_003098] | -3.03 | 2.43E-03 |
| A\_32\_P110372 | SLC8A1 | solute carrier family 8 (sodium/calcium exchanger), member 1 [NM\_021097] | -3.03 | 8.81E-03 |
| A\_32\_P185229 | ATP8B2 | ATPase, Class I, type 8B, member 2 [NM\_020452] | -3.03 | 6.57E-04 |
| A\_23\_P123018 | WBSCR23 | Williams-Beuren syndrome chromosome region 23 [NM\_025042] | -3.03 | 2.10E-02 |
| A\_24\_P267619 | PTPNS1 | protein tyrosine phosphatase, non-receptor type substrate 1 [NM\_080792] | -2.94 | 1.78E-02 |
| A\_24\_P383277 | KIAA1715 | Homo sapiens KIAA1715 [NM\_030650] | -2.94 | 3.81E-02 |
| A\_23\_P355447 | ZDHHC22 | zinc finger, DHHC domain containing 22 [NM\_174976] | -2.94 | 1.19E-02 |
| A\_24\_P361816 | IGLV6-57 | immunoglobulin lambda variable 6-57 [AF267874] | -2.94 | 2.43E-03 |
| A\_24\_P379693 | DNPEP | aspartyl aminopeptidase. [AK023005] | -2.94 | 1.30E-02 |
| A\_24\_P279704 | MAG | myelin associated glycoprotein [NM\_080600] | -2.86 | 4.26E-10 |
| A\_23\_P142075 | ACP5 | acid phosphatase 5, tartrate resistant [NM\_001611] | -2.86 | 1.89E-03 |
| A\_32\_P75902 | C16orf73 | chromosome 16 open reading frame 73 [NM\_152764] | -2.86 | 4.74E-02 |
| A\_23\_P130653 | RTBDN | retbindin [NM\_031429] | -2.86 | 3.24E-03 |
| A\_23\_P109201 | C20orf3 | chromosome 20 open reading frame 3 [NM\_020531] | -2.86 | 7.20E-03 |
| A\_23\_P253982 | HOXA4 | homeo box A4 [NM\_002141] | -2.78 | 1.11E-02 |
| A\_23\_P117082 | HEBP1 | heme binding protein 1 [NM\_015987] | -2.78 | 9.49E-03 |
| A\_23\_P30913 | HLA-DPA1 | major histocompatibility complex, class II, DP alpha 1 [NM\_033554] | -2.78 | 3.24E-03 |
| A\_23\_P379630 | SLC38A10 | solute carrier family 38, member 10 [NM\_138570] | -2.78 | 1.99E-02 |
| A\_32\_P134657 | KIAA0240 | Homo sapiens KIAA0240 (KIAA0240), mRNA [NM\_015349] | -2.70 | 1.26E-02 |

## Slide 4
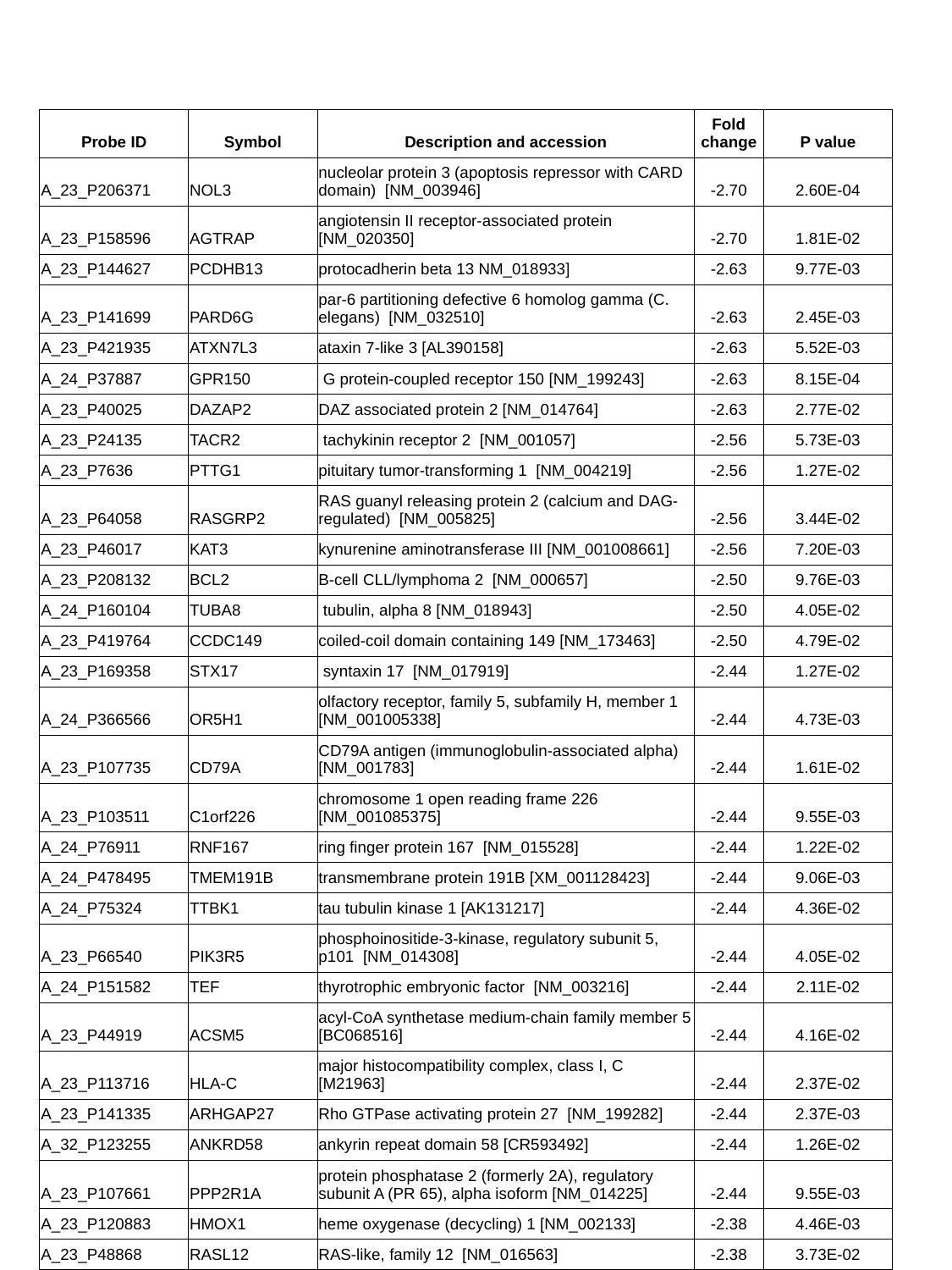

| Probe ID | Symbol | Description and accession | Fold change | P value |
| --- | --- | --- | --- | --- |
| A\_23\_P206371 | NOL3 | nucleolar protein 3 (apoptosis repressor with CARD domain) [NM\_003946] | -2.70 | 2.60E-04 |
| A\_23\_P158596 | AGTRAP | angiotensin II receptor-associated protein [NM\_020350] | -2.70 | 1.81E-02 |
| A\_23\_P144627 | PCDHB13 | protocadherin beta 13 NM\_018933] | -2.63 | 9.77E-03 |
| A\_23\_P141699 | PARD6G | par-6 partitioning defective 6 homolog gamma (C. elegans) [NM\_032510] | -2.63 | 2.45E-03 |
| A\_23\_P421935 | ATXN7L3 | ataxin 7-like 3 [AL390158] | -2.63 | 5.52E-03 |
| A\_24\_P37887 | GPR150 | G protein-coupled receptor 150 [NM\_199243] | -2.63 | 8.15E-04 |
| A\_23\_P40025 | DAZAP2 | DAZ associated protein 2 [NM\_014764] | -2.63 | 2.77E-02 |
| A\_23\_P24135 | TACR2 | tachykinin receptor 2 [NM\_001057] | -2.56 | 5.73E-03 |
| A\_23\_P7636 | PTTG1 | pituitary tumor-transforming 1 [NM\_004219] | -2.56 | 1.27E-02 |
| A\_23\_P64058 | RASGRP2 | RAS guanyl releasing protein 2 (calcium and DAG-regulated) [NM\_005825] | -2.56 | 3.44E-02 |
| A\_23\_P46017 | KAT3 | kynurenine aminotransferase III [NM\_001008661] | -2.56 | 7.20E-03 |
| A\_23\_P208132 | BCL2 | B-cell CLL/lymphoma 2 [NM\_000657] | -2.50 | 9.76E-03 |
| A\_24\_P160104 | TUBA8 | tubulin, alpha 8 [NM\_018943] | -2.50 | 4.05E-02 |
| A\_23\_P419764 | CCDC149 | coiled-coil domain containing 149 [NM\_173463] | -2.50 | 4.79E-02 |
| A\_23\_P169358 | STX17 | syntaxin 17 [NM\_017919] | -2.44 | 1.27E-02 |
| A\_24\_P366566 | OR5H1 | olfactory receptor, family 5, subfamily H, member 1 [NM\_001005338] | -2.44 | 4.73E-03 |
| A\_23\_P107735 | CD79A | CD79A antigen (immunoglobulin-associated alpha) [NM\_001783] | -2.44 | 1.61E-02 |
| A\_23\_P103511 | C1orf226 | chromosome 1 open reading frame 226 [NM\_001085375] | -2.44 | 9.55E-03 |
| A\_24\_P76911 | RNF167 | ring finger protein 167 [NM\_015528] | -2.44 | 1.22E-02 |
| A\_24\_P478495 | TMEM191B | transmembrane protein 191B [XM\_001128423] | -2.44 | 9.06E-03 |
| A\_24\_P75324 | TTBK1 | tau tubulin kinase 1 [AK131217] | -2.44 | 4.36E-02 |
| A\_23\_P66540 | PIK3R5 | phosphoinositide-3-kinase, regulatory subunit 5, p101 [NM\_014308] | -2.44 | 4.05E-02 |
| A\_24\_P151582 | TEF | thyrotrophic embryonic factor [NM\_003216] | -2.44 | 2.11E-02 |
| A\_23\_P44919 | ACSM5 | acyl-CoA synthetase medium-chain family member 5 [BC068516] | -2.44 | 4.16E-02 |
| A\_23\_P113716 | HLA-C | major histocompatibility complex, class I, C [M21963] | -2.44 | 2.37E-02 |
| A\_23\_P141335 | ARHGAP27 | Rho GTPase activating protein 27 [NM\_199282] | -2.44 | 2.37E-03 |
| A\_32\_P123255 | ANKRD58 | ankyrin repeat domain 58 [CR593492] | -2.44 | 1.26E-02 |
| A\_23\_P107661 | PPP2R1A | protein phosphatase 2 (formerly 2A), regulatory subunit A (PR 65), alpha isoform [NM\_014225] | -2.44 | 9.55E-03 |
| A\_23\_P120883 | HMOX1 | heme oxygenase (decycling) 1 [NM\_002133] | -2.38 | 4.46E-03 |
| A\_23\_P48868 | RASL12 | RAS-like, family 12 [NM\_016563] | -2.38 | 3.73E-02 |
| A\_23\_P388900 | SLC22A15 | solute carrier family 22 (organic cation transporter), member 15 [NM\_018420] | -2.38 | 7.77E-03 |
| A\_23\_P61633 | TNK2 | tyrosine kinase, non-receptor, 2 [NM\_005781] | -2.38 | 4.96E-05 |
| A\_23\_P159927 | ARHGAP4 | Rho GTPase activating protein 4 [NM\_001666] | -2.38 | 7.28E-03 |
| A\_23\_P78808 | SYMPK | symplekin [NM\_004819] | -2.38 | 9.94E-03 |
| A\_23\_P109864 | GHSR | growth hormone secretagogue receptor [NM\_004122] | -2.38 | 9.76E-03 |
| A\_24\_P166443 | HLA-DPB1 | major histocompatibility complex, class II, DP beta 1 [NM\_002121] | -2.38 | 2.43E-02 |
| A\_23\_P117852 | KIAA0101 | KIAA0101 [NM\_014736] | -2.38 | 1.89E-02 |
| A\_23\_P107369 | KIF1C | kinesin family member 1C [NM\_006612] | -2.38 | 2.61E-03 |
| A\_24\_P38895 | H2AFX | H2A histone family, member X [NM\_002105] | -2.33 | 1.34E-02 |
| A\_24\_P102895 | WDTC1 | WD and tetratricopeptide repeats 1 [NM\_015023] | -2.33 | 3.34E-03 |

## Slide 5
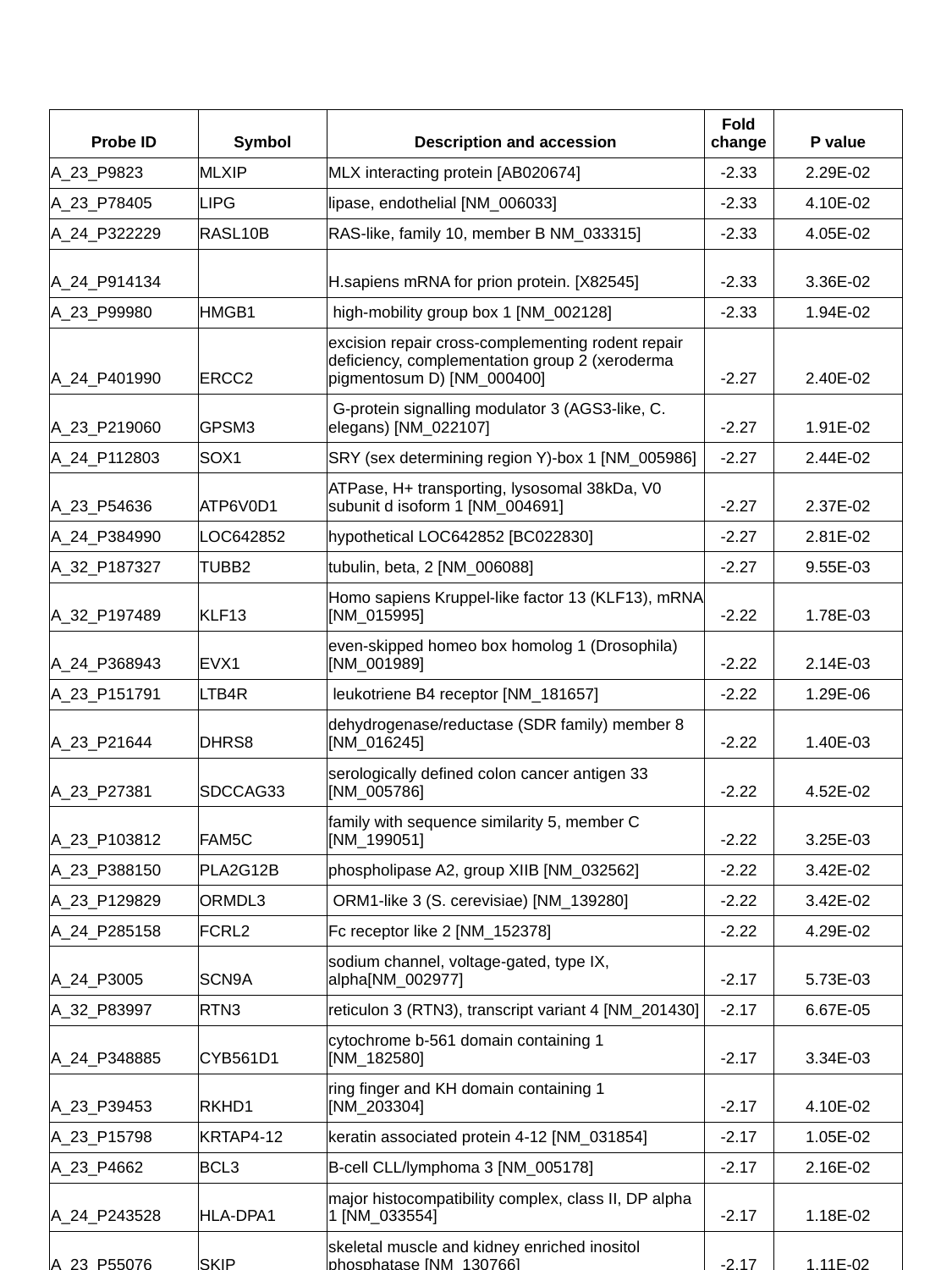

| Probe ID | Symbol | Description and accession | Fold change | P value |
| --- | --- | --- | --- | --- |
| A\_23\_P9823 | MLXIP | MLX interacting protein [AB020674] | -2.33 | 2.29E-02 |
| A\_23\_P78405 | LIPG | lipase, endothelial [NM\_006033] | -2.33 | 4.10E-02 |
| A\_24\_P322229 | RASL10B | RAS-like, family 10, member B NM\_033315] | -2.33 | 4.05E-02 |
| A\_24\_P914134 | | H.sapiens mRNA for prion protein. [X82545] | -2.33 | 3.36E-02 |
| A\_23\_P99980 | HMGB1 | high-mobility group box 1 [NM\_002128] | -2.33 | 1.94E-02 |
| A\_24\_P401990 | ERCC2 | excision repair cross-complementing rodent repair deficiency, complementation group 2 (xeroderma pigmentosum D) [NM\_000400] | -2.27 | 2.40E-02 |
| A\_23\_P219060 | GPSM3 | G-protein signalling modulator 3 (AGS3-like, C. elegans) [NM\_022107] | -2.27 | 1.91E-02 |
| A\_24\_P112803 | SOX1 | SRY (sex determining region Y)-box 1 [NM\_005986] | -2.27 | 2.44E-02 |
| A\_23\_P54636 | ATP6V0D1 | ATPase, H+ transporting, lysosomal 38kDa, V0 subunit d isoform 1 [NM\_004691] | -2.27 | 2.37E-02 |
| A\_24\_P384990 | LOC642852 | hypothetical LOC642852 [BC022830] | -2.27 | 2.81E-02 |
| A\_32\_P187327 | TUBB2 | tubulin, beta, 2 [NM\_006088] | -2.27 | 9.55E-03 |
| A\_32\_P197489 | KLF13 | Homo sapiens Kruppel-like factor 13 (KLF13), mRNA [NM\_015995] | -2.22 | 1.78E-03 |
| A\_24\_P368943 | EVX1 | even-skipped homeo box homolog 1 (Drosophila)[NM\_001989] | -2.22 | 2.14E-03 |
| A\_23\_P151791 | LTB4R | leukotriene B4 receptor [NM\_181657] | -2.22 | 1.29E-06 |
| A\_23\_P21644 | DHRS8 | dehydrogenase/reductase (SDR family) member 8 [NM\_016245] | -2.22 | 1.40E-03 |
| A\_23\_P27381 | SDCCAG33 | serologically defined colon cancer antigen 33 [NM\_005786] | -2.22 | 4.52E-02 |
| A\_23\_P103812 | FAM5C | family with sequence similarity 5, member C [NM\_199051] | -2.22 | 3.25E-03 |
| A\_23\_P388150 | PLA2G12B | phospholipase A2, group XIIB [NM\_032562] | -2.22 | 3.42E-02 |
| A\_23\_P129829 | ORMDL3 | ORM1-like 3 (S. cerevisiae) [NM\_139280] | -2.22 | 3.42E-02 |
| A\_24\_P285158 | FCRL2 | Fc receptor like 2 [NM\_152378] | -2.22 | 4.29E-02 |
| A\_24\_P3005 | SCN9A | sodium channel, voltage-gated, type IX, alpha[NM\_002977] | -2.17 | 5.73E-03 |
| A\_32\_P83997 | RTN3 | reticulon 3 (RTN3), transcript variant 4 [NM\_201430] | -2.17 | 6.67E-05 |
| A\_24\_P348885 | CYB561D1 | cytochrome b-561 domain containing 1 [NM\_182580] | -2.17 | 3.34E-03 |
| A\_23\_P39453 | RKHD1 | ring finger and KH domain containing 1 [NM\_203304] | -2.17 | 4.10E-02 |
| A\_23\_P15798 | KRTAP4-12 | keratin associated protein 4-12 [NM\_031854] | -2.17 | 1.05E-02 |
| A\_23\_P4662 | BCL3 | B-cell CLL/lymphoma 3 [NM\_005178] | -2.17 | 2.16E-02 |
| A\_24\_P243528 | HLA-DPA1 | major histocompatibility complex, class II, DP alpha 1 [NM\_033554] | -2.17 | 1.18E-02 |
| A\_23\_P55076 | SKIP | skeletal muscle and kidney enriched inositol phosphatase [NM\_130766] | -2.17 | 1.11E-02 |
| A\_23\_P363196 | TCL6 | T-cell leukemia/lymphoma 6 [NM\_014418] | -2.17 | 4.43E-03 |
| A\_24\_P21715 | RAD9A | RAD9 homolog A (S. pombe) [NM\_004584] | -2.13 | 7.28E-03 |
| A\_24\_P355609 | MRGPRF | MAS-related GPR, member F [NM\_145015] | -2.13 | 2.22E-02 |
| A\_23\_P422922 | TAF3 | TAF3 RNA polymerase II, TATA box binding protein (TBP)-associated factor, 140kDa [BC073884] | -2.13 | 1.94E-02 |
| A\_23\_P371794 | CACNB3 | calcium channel, voltage-dependent, beta 3 subunit [NM\_000725] | -2.13 | 2.94E-02 |
| A\_23\_P255884 | GSN | gelsolin (amyloidosis, Finnish type) [NM\_198252] | -2.08 | 2.54E-02 |
| A\_32\_P207124 | CT47 | cancer/testis CT47 family, member 11 [NM\_173571] | -2.08 | 2.10E-02 |
| A\_23\_P60324 | UBADC1 | ubiquitin associated domain containing 1 [NM\_016172] | -2.08 | 1.36E-02 |
| A\_23\_P207842 | RARA | retinoic acid receptor, alpha [NM\_000964] | -2.08 | 3.24E-03 |

## Slide 6
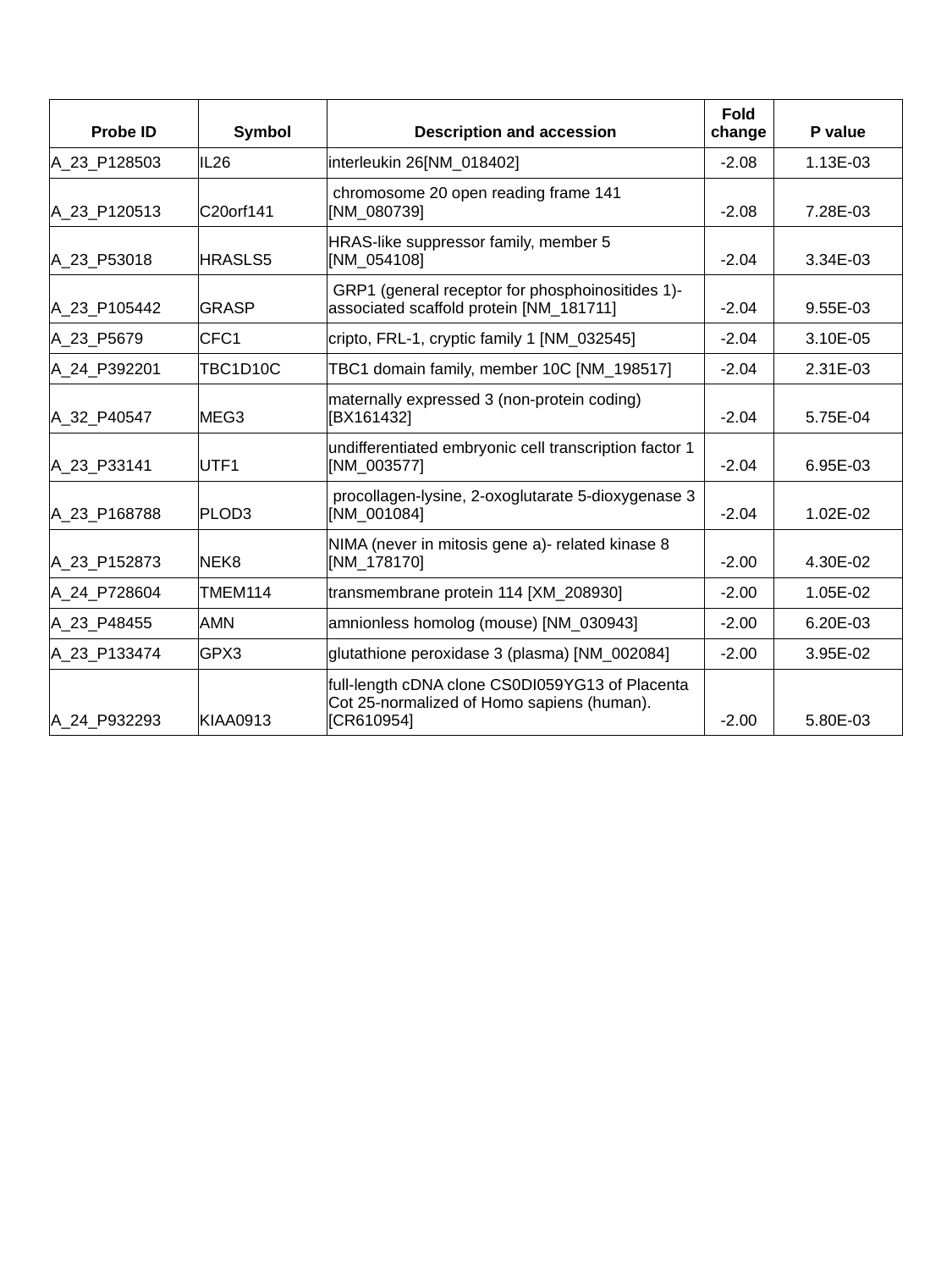

| Probe ID | Symbol | Description and accession | Fold change | P value |
| --- | --- | --- | --- | --- |
| A\_23\_P128503 | IL26 | interleukin 26[NM\_018402] | -2.08 | 1.13E-03 |
| A\_23\_P120513 | C20orf141 | chromosome 20 open reading frame 141 [NM\_080739] | -2.08 | 7.28E-03 |
| A\_23\_P53018 | HRASLS5 | HRAS-like suppressor family, member 5 [NM\_054108] | -2.04 | 3.34E-03 |
| A\_23\_P105442 | GRASP | GRP1 (general receptor for phosphoinositides 1)-associated scaffold protein [NM\_181711] | -2.04 | 9.55E-03 |
| A\_23\_P5679 | CFC1 | cripto, FRL-1, cryptic family 1 [NM\_032545] | -2.04 | 3.10E-05 |
| A\_24\_P392201 | TBC1D10C | TBC1 domain family, member 10C [NM\_198517] | -2.04 | 2.31E-03 |
| A\_32\_P40547 | MEG3 | maternally expressed 3 (non-protein coding) [BX161432] | -2.04 | 5.75E-04 |
| A\_23\_P33141 | UTF1 | undifferentiated embryonic cell transcription factor 1 [NM\_003577] | -2.04 | 6.95E-03 |
| A\_23\_P168788 | PLOD3 | procollagen-lysine, 2-oxoglutarate 5-dioxygenase 3 [NM\_001084] | -2.04 | 1.02E-02 |
| A\_23\_P152873 | NEK8 | NIMA (never in mitosis gene a)- related kinase 8 [NM\_178170] | -2.00 | 4.30E-02 |
| A\_24\_P728604 | TMEM114 | transmembrane protein 114 [XM\_208930] | -2.00 | 1.05E-02 |
| A\_23\_P48455 | AMN | amnionless homolog (mouse) [NM\_030943] | -2.00 | 6.20E-03 |
| A\_23\_P133474 | GPX3 | glutathione peroxidase 3 (plasma) [NM\_002084] | -2.00 | 3.95E-02 |
| A\_24\_P932293 | KIAA0913 | full-length cDNA clone CS0DI059YG13 of Placenta Cot 25-normalized of Homo sapiens (human). [CR610954] | -2.00 | 5.80E-03 |
