# Supplementary material for: Osteosarcoma is characterised by reduced expression of markers of osteoclastogenesis and antigen presentation compared with normal bone
Source: Br J Cancer. 2010 Jun 15;103(1):73–81. doi: 10.1038/sj.bjc.6605723 (PMC2905286; doi:10.1038/sj.bjc.6605723)
Supplement: Supplementary Materials and Methods [file 6605723x2.doc]

**Endo-Munoz et al.**

**SUPPLEMENTARY MATERIALS AND METHODS**

#### Tissue culture and cell lines

Human dermal fibroblasts (HDF), squamous cell carcinoma cells (COLO-16), prostate carcinoma cells (LNCap, DU145), ovarian cancer cells (DOV13), breast cancer cells (SK-BR3), osteosarcoma cells (U2OS, HOS and KHOS) and colon adenocarcinoma cells (HT29) were cultured in Dulbecco’s Modified Eagle Medium (DMEM) containing 10% fetal calf serum (FCS) at 37◦C in 5% CO_2_ until confluence. The Burkitt lymphoma cell line DG75 was grown in RPMI 1640/10% FCS to 2.2 x 10^6^ cells/mL.

**RNA extraction**

RNA integrity was confirmed by formaldehyde agarose gel electrophoresis and by PCR using β-actin primers: 5'- GGA CCT GAC TGA CTA CCT CA - 3' and 5'- AGC TTC TCC - 3'.

**Reverse transcription**

At least 20 μg of tumour, non-malignant or reference RNA were incubated with anchored oligo(dT)12-18 primer (Invitrogen). Reverse transcription was then performed with 0.65 μL of aminoallyl dNTP - 25 mM dATP, 25 mM dGTP, 25mM dCTP, 7.5 mM dTTP (Invitrogen) and 15 mM aminoallyl d-UTP (Sigma, St. Louis, MO), and 400 U of Superscript II (Invitrogen). Purification of the amine-modified cDNA was performed using a QIAquick PCR Purification Kit (Qiagen, Hilden, Germany).

**Reference RNA panel and microarray analysis**

RNA from each of the eight cell lines was diluted to equal concentrations, pooled, aliquoted and stored at -80◦C. Labeling of tumour, non-malignant bone, and reference cDNA was performed with AlexaFluor 555 or AlexaFluor 647 (Invitrogen).

**Data analysis**

Spot intensities were corrected for background and flagged as present or absent by filtering spots that were missing, had low signal or were of poor quality. For each sample, signal median intensity and standard deviation measurements for each dye were exported to Microsoft Excel. Median intensities for the *Arabidopsis* controls on the human array were used to discard human genes that had median intensities lower than the average median intensity +/- 3SD of control genes. Per spot and per chip intensity-dependent (LOWESS) normalization was applied to the data. The normalized intensity measurements from each experiment were log transformed (base 2) and the log ratio of experimental *vs.* reference channel was used as the measure of gene expression. The normalized log ratio of the data from genes flagged as present in at least 50% of samples was analyzed further by applying the cross gene error model (CGEM) to identify genes for which the signal strength was consistently high in order to be considered high trust. These genes were then filtered to include those whose expression was up- or down-regulated by at least 2-fold. These genes were analyzed using a one-way ANOVA Welch *t*-test for unpaired comparison of each of the two groups: osteosarcoma *vs*. non-malignant bone, and good responders *vs.* poor responders, with a *P*-value cutoff of 0.05 and multiple testing correction (Benjamini and Hochberg False Discovery Rate) to define a set of significantly up- and downregulated genes. Unsupervised hierarchical clustering was performed on the differentially expressed genes between good and poor responders using standard correlation as a similarity measure. Genes were annotated and biological processes were analyzed by Ingenuity pathway analysis (IPA; Ingenuity Systems, [https://www.ingenuity.com](https://www.ingenuity.com/)).

**ACP5/TRAP staining and immunohistochemistry.**

Immunohistochemistry of formalin-fixed paraffin-embedded (FFPE) sections was performed with mouse monoclonal antibody to human tartrate-resistance acid phosphatase (ACP5/TRAP) Ab-1 (clone 26E5) (NeoMarkers, Freemont, CA) according to manufacturer’s instructions. Negative controls used appropriate mouse or rabbit IgG (DakoCytomation, Denmark). Detection and staining was achieved with the Starr Trek Universal HRP Detection and Cardassian DAB Chromogen Substrate Kits (Biocare Medical, Concord, CA). The number of osteoclasts was quantified by counting the number of ACP5/TRAP^+ve^ cells per field in 9-16 microscope fields (20X).
